# Study on Mechanical Properties and Carbon Emission Analysis of Polypropylene Fiber-Reinforced Brick Aggregate Concrete

**DOI:** 10.3390/polym16243535

**Published:** 2024-12-19

**Authors:** Fei Li, Shenghao Jin, Peifeng Cheng, Zehui Wang, Zehao Yang

**Affiliations:** 1 Changda Construction Technology Co., Ltd., Weifang 261205, China; xiaofei2724@163.com; 2 School of Civil Engineering and Transportation, Northeast Forestry University, Harbin 150040, China; shenghao.jin@nefu.edu.cn (S.J.); 13516349060@163.com (Z.W.); 15524232871@163.com (Z.Y.)

**Keywords:** fiber recycled concrete, recycled brick aggregate, polypropylene fiber, mechanical property, intensity conversion relation, carbon emission assessment

## Abstract

Given the current construction waste accumulation problem, to utilize the resource of red brick solid waste, construction waste red brick was used as a concrete coarse aggregate combined with polypropylene fiber to prepare PPF (polypropylene fiber)-reinforced recycled brick aggregate concrete. Through a cube compression test, axial compression test, and four-point bending test of 15 groups of specimens, the influences of the aggregate replacement rate of recycled brick and the PPF volume on the mechanical properties of recycled brick aggregate concrete reinforced by PPF were studied, and a strength parameter calculation formula was constructed and modified based on the above. Finally, combined with a life cycle assessment (LCA), the carbon emissions of raw materials were analyzed and evaluated. It was found that the mechanical properties of recycled concrete enhanced by PPF are critical at an addition rate of 50% and then decrease slowly with an increase in the aggregate content. PPF effectively alleviates the problem of strength reductions caused by regenerated aggregate substitution through the fiber-bridging effect. Based on the experimental data, a mechanical transformation model considering fiber reinforcement and BA weakening was constructed, and the regression accuracy R2 was around 0.90. The environmental benefit obtained when only replacing the natural aggregate is low. Although the incorporation of fiber improves the carbon emissions of the material to a certain extent, the benefits are more noticeable compared with the increase in strength. The results show that garbage recovery and strength demand benefits are achieved when the amount of recycled brick aggregate is 50% of the total. The strength conversion model established in this paper has of high accuracy and was created with careful consideration of fiber reinforcement and the regenerated aggregate weakening correction, providing it with more robust adaptability and extensibility. The mechanical properties of the recycled brick aggregate concrete enhanced by PPF are excellent and sustainable when the replacement rate of BA is 50% and the PPF volume is 0.1%.

## 1. Introduction

The resource utilization of solid waste is becoming a topic of increasing interest. Industrial solid waste and construction solid waste constitute the most significant components of solid waste. Regarding solid industrial waste (fly ash and slag), the current approach to resource utilization mainly involves preparing geopolymers via alkali excitation to replace the cementing materials in concrete and achieve a high-value utilization of waste [1,2]. The most common utilization of solid construction waste at present lies in preparing it for use as a concrete aggregate.

With the acceleration of urbanization, many old buildings will be demolished, inevitably producing a large amount of construction waste. Construction waste mainly includes waste concrete and waste bricks, of which waste bricks account for 35%~45% [3]. Recycled brick aggregate (BA) is made from waste fired brick after crushing, screening, cleaning, and drying, and the natural aggregate is partially or completely replaced by this to prepare recycled brick aggregate concrete (BAC), which is one of the effective ways of utilizing masonry construction waste [4]. The recycling and reuse of BA reduce the consumption of natural stone and reduce the status quo of construction waste accumulation, conforming to the current theme of “green, low-carbon, and environmental protection”.

Compared with natural aggregates, BA prepared from waste brick has terrible characteristics, such as high water absorption, a high pressure crushing value, and low apparent density, which seriously affect the compressive strength of BAC [5,6]. Many experts and scholars have researched the recycling and utilization of waste-sintered bricks. Mohammed et al. [6] collected raw materials from 33 construction sites in different locations and crushed them into recycled brick aggregate to prepare more than 700 cylindrical concrete samples. By conducting experiments on the mechanical properties of samples of different ages, the feasibility of obtaining concrete with a strength of 20.7MPa~31.0 MPa was verified, and the optimal water–binder ratio was found to be 0.45. Lan et al. [7] conducted a study on the compressive strength of recycled concrete with different aggregate substitution rates; the results showed that the compressive strength of recycled concrete decreased with an increase in aggregate substitution rates. Liu et al. [8] tested the mechanical properties of three groups of recycled aggregate concrete of different grades, verified the influence of recycled aggregate particle grade on the strength of recycled concrete, and built a calculation model of the mechanical parameters of the three groups of recycled concrete.

The above research explored the effect of the BA substitution rate on BAC’s mechanical properties and whether the compressive strength of recycled concrete could be enhanced by using gradation optimization or aggregate strengthening. However, the improvement in the strength of BAC when using a single optimized aggregate grade is limited. When a certain proportion of various fibers (basalt fiber [9], steel fiber [10,11], polypropylene fiber [12], and polyvinyl alcohol fiber [13,14]) are incorporated into BAC, the defects in BAC can be improved through the toughening effect of the fibers and the bridging effect to broaden its application range [15,16,17]. Mansour et al. [18] analyzed the influence of different steel fiber contents on the flexural strength of recycled concrete and proposed a reasonable value of 2% steel fiber content. Chaboki et al. [19] studied how adding steel fiber effectively inhibits the generation and propagation of cracks in recycled concrete beams. Weal et al. [20] took the basalt fiber content and recycled aggregate substitution rate as variables to test the mechanical properties of fiber-recycled concrete and found that compared with the recycled aggregate substitution rate, the fiber incorporation rate had a more significant impact on the strength and deformation resistance of BAC. Fayed et al. [21] explored the size effect of steel fiber-reinforced recycled concrete under different replacement levels of recycled aggregate and different steel fiber contents and established a bearing capacity prediction formula. Based on the above, the synergistic effect of steel fiber and polypropylene fiber on RC columns was further explored [22]. The addition of fiber can effectively improve the mechanical properties of recycled concrete and alleviate the weakening caused by aggregate defects. The above research results provide a solid foundation for research on the mechanical parameter conversion of PPRAC (polypropylene fiber reinforced brick aggregate recycled concrete) presented in this paper.

In summary, scholars have mainly studied the mechanical properties of steel fiber/polypropylene fiber/basalt fiber/polypropylene alcohol fiber recycled concrete, obtaining relatively fruitful results. Compared with previous studies, the aims of this paper are to establish the mechanical parameter conversion relationship of polypropylene fiber recycled concrete with higher precision and to balance the environmental benefits and mechanical index to suggest a reasonable ratio.

In order to explore the synergistic effect of BA and PP (polypropylene fiber) and provide a reference for the application of PPRAC in various projects, this paper takes the BA substitution rate and PP content as parameters. Fifteen experimental groups with BA substitution rates of 0, 25%, 50%, 75%, and 100% and PP contents of 0, 0.1%, and 0.2% were used in a cube compression test, axial compression test, and flexural strength test. The mechanical strength and elastic modulus calculation formulas of PPRAC were analyzed and established. Finally, the life cycle assessment (LCA) method calculates the carbon emission from the production end of the raw material. In this paper, the recommended value of the two indexes is given.

## 2. Materials and Methods

### 2.1. Materials and Properties

**Figure 1:**
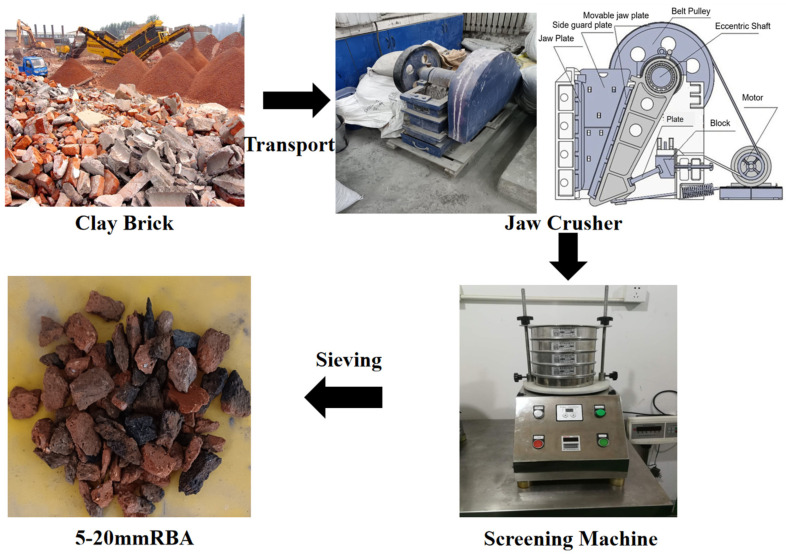
Progress flow of recycled brick aggregate.

**Figure 2:**
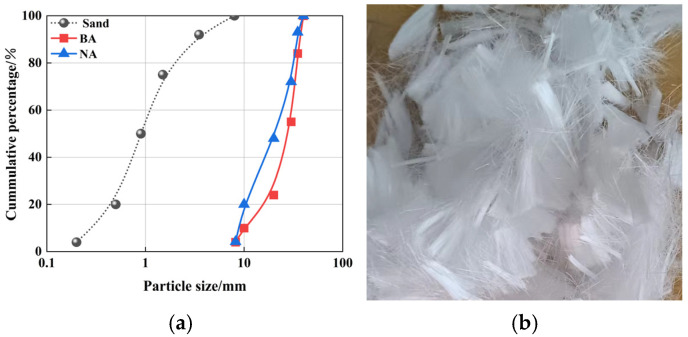
Fiber recycled concrete main material: (**a**) grading of coarse and fine aggregate; (**b**) 15 mm PPF.

The waste brick material utilized in this study this study was obtained from the demolition of old houses that had been in use for approximately 45 years; the brick material was clay brick. Following transportation from the demolition site to the laboratory, the bricks were processed using a jaw crusher, followed by sieving, cleaning, and drying. The specific preparation process is shown in Figure 1. The particle size gradation of coarse and fine aggregates used in the study is shown in Figure 2a. Given that the particle size distribution of recycled brick aggregates closely resembles that of natural aggregates, it was only necessary to make an equivalent substitution in volume when designing the mix ratio. The mechanical properties of BA and NA are shown in Table 1. Polypropylene fiber (PPF), as depicted in Figure 2b, along with its mechanical property parameters, are detailed in Table 2.

In this experiment, the test specimens mainly consisted of coarse aggregate, fine aggregate, fiber, and cement. The cement used in the test specimens was P·O 42.5 ordinary Portland cement from Harbin Cement Plant. Its main components and characteristics are shown in Table 3 and Table 4. The fine aggregate used in this paper was natural medium sand. Figure 2a presented the particle size grading curve, while other physical characteristics, obtained according to JGJ 52-2006 [23], are presented in Table 5.

### 2.2. Mixing Ratio Design and Specimen Preparation

By selecting the PPF content and BA substitution rate as key indexes, 15 concrete groups were designed and tested, with a target compressive strength of C30. The PPF volume content was categorized into 0, 0.1%, 0.15% levels, and the BA replacement rate was set at 0, 25%, 50%, 75%, and 100%. The corresponding RA proportion was 100%, 75%, 50%, 25%, and 0. Fifteen groups of specimens were numbered successively; their specific numbers and mix ratios are detailed in Table 6 and Table 7. Among them, 1-x, 2-x, and 3-x represent the groups with a PPF contents of 0%, 0.10%, and 0.15%, respectively. x-1, x-2, x-3, x-4, and x-5 represent the replacement rates of recycled brick aggregate at 0, 25%, 50%, 75%, and 100%, respectively.

Nine cube specimens with a side length of 100 mm were prepared for each group of mix ratio to carry out cube compression tests. Three cylindrical specimens with a bottom diameter of 100 mm and a height of 200 mm were used in axial compression tests, and three prismatic specimens with a side length of 100 mm and a height of 400 mm were used in bending tests. The preparation process of the specimen involved wetting the inner wall of the mixer and waiting for it to dry slightly before adding the material. We then weighed the material according to the mix ratio in Table 7.

The sample preparation process of PPRAC was as follows: (1) BA and sand was poured into the mixer and stirred for 60 s. Then, we added cement and water and stirred for 30 s. (2) PPF was added twice to stir the mixture for 60 s. (3) The other half of the water was added to achieve the desired stirring state. Then, we took the mixture out, put it into the test mold, and put it on a vibration table for vibration compaction. (4) After standing for 24 h, the film was removed and placed in a standard curing room for 28 days (temperature: 20 ± 2 °C; relative humidity: >95%).

BA was soaked in water for 24 h before pouring to ensure that the surface was saturated and dry to eliminate the adverse effects of water absorption on the amount of water required for mixing.

### 2.3. Testing Methods

We conducted uniaxial compression strength tests on cylindrical and cubic specimens and four-point bending tests on prismatic specimens. The microcomputer-controlled electro-hydraulic servo universal testing machine used in this experiment was manufactured by Shenzhen Gongyouji Group Co., Ltd. (Shenzhen, China), with a maximum static load capacity is 1000 kN.

**Figure 3:**
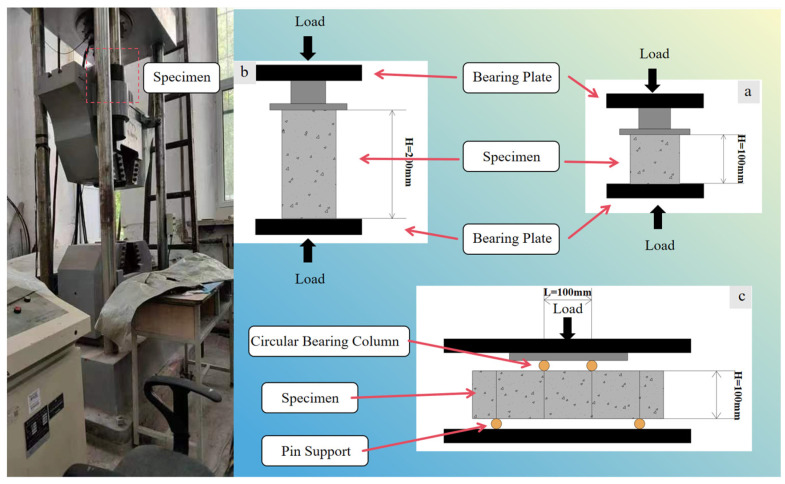
Mechanical property test: (**a**) Cube compression test; (**b**) Axial compression test; (**c**) Four-point bending test.

According to GB/T 5008-2019 [24], the compressive strength of concrete was tested, as shown in Figure 3a,b. The standard test was conducted to obtain the specimens’ cubic compressive strength and axial compressive strength. The loading system complied with the standard requirements with a loading speed of 0.6 MPa/s. Among them, the curing age of the specimens used for the cube compression test was 7 d, 28 d, and the curing age of the specimens used for the axial compression test was 28 d.

In order to determine the flexural strength of PPRAC containing recycled brick aggregate after the first crack, this study conducted bending tests on prismatic concrete, as shown in Figure 3c. This method is simple and less sensitive to boundary conditions. The loading process complied with the CNS 1223-1984 standard [25]. Each group’s curing age of PPRAC was 28 days, and the average flexural strength of 100 × 100 × 400 mm^3^ prisms of the exact group specifications and test methods was selected as the bending strength. The bending strength can be calculated according to *F*_b_ = *P*_max_ · *l*/(*bh*^2^), where *F*_b_ is the bending strength, *P*_max_ is the peak load, *l* is the span (300 mm), *b* is the width, and *h* is the height.

## 3. Experimental Results and Discussion

### 3.1. Compressive Strength of Cube

**Figure 4:**
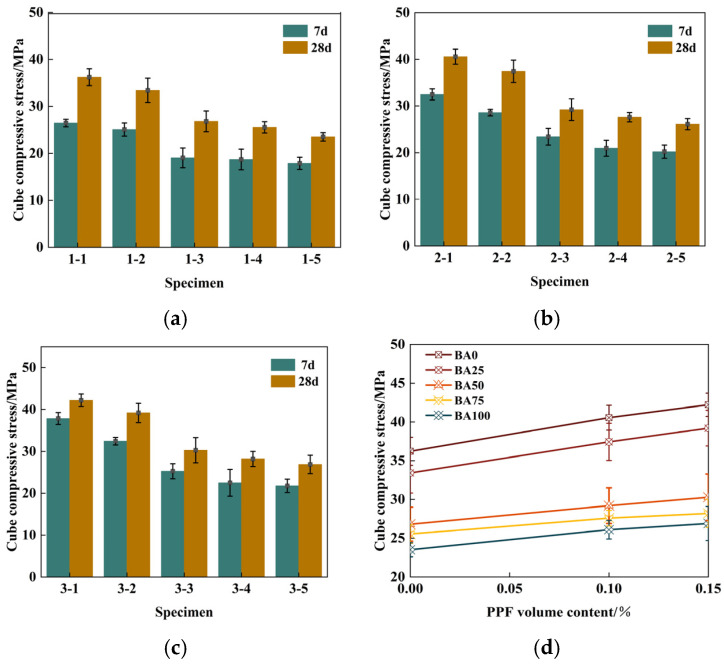
PPF recycled brick aggregate concrete cube compressive strength: (**a**) Effects of BA content (0 PPF); (**b**) Effects of BA content (0.1%PPF); (**c**) Effects of BA content (0.15%PPF); (**d**) Effects of PPF content.

Cube compressive strength is an essential index for evaluating concrete. Figure 4a–c shows the influence of the BA substitution rate on the compressive strength of recycled brick aggregate concrete at different ages when the PPF volume content was 0, 0.1%, and 0.15%. It can be observed that in the three fiber content groups, 1-1, 2-1, and 3-1, the control group showed the most considerable 28-day compressive strength; the compressive strength values were 36.21 MPa, 40.56 MPa, and 42.22 MPa, respectively. The 7 d and 28 d age bars show a downward trend, but there was a rapid deterioration at the 25–50% BA incorporation stage. Due to the roughness of the surface of brick aggregate and the high porosity, both of these characteristics contributed to the enhancement of interface adhesion. In addition, the hydration product of the cement slurry entered the pores of the brick aggregate after precipitation and was nested with the aggregate, thus improving the mechanical properties of the recycled brick aggregate concrete. However, with the addition of recycled brick aggregate reaching 50%, the nesting effect could no longer offset the strength loss caused by the aggregate defects, so the strength of recycled brick aggregate concrete decreased sharply. When the aggregate content was 25%, the compressive strength of the recycled concrete cubes in the three fiber content groups was reduced by 7.7%, 7.8%, and 11.4%, respectively. When the aggregate content increased to 50%, the strength loss rate rapidly increased to 19.8%, 21.9%, and 24.3%. Therefore, only from the perspective of cube compressive strength should the amount of RB incorporation be at most 50%.

Figure 4d shows the influence of different fiber contents on the strength of recycled brick aggregate concrete. It can be observed that the strength of PPRAC increased with an increase in PPF content, but the growth trend slowed. The 28 d strength of recycled concrete mixed with 0.1% PPF increased by 12.01%, 11.98%, 9.16%, 8.07%, and 11.01%, and the overall improvement range was between 8% and 13%. Compared with the experimental group containing 0.1% PPF in the control group, the 28 d intensity increased by 4.59%, 4.72%, 3.70%, 2.17%, and 3.07%, and the overall improvement range was between 2% and 5%. This was due to the bridge effect of PPF [23,24], which alleviated the negative impact of the addition of BA on the compressive strength of the cube. When PPF was incorporated into recycled concrete, it functioned similarly to the distribution of several small steel bars, i.e., bearing the tensile stress at cracks, which enhanced the compressive strength of the concrete. At the same time, the microstructure of concrete specimens was improved, and the concrete was compact. However, the excessive PPF appeared to be a clumping phenomenon when the volume of PPF increased to 0.15%, which could not give full play to the bridging effect, resulting in a decrease in the intensity range. The average value of adding 0.1% PPF to the 7 d strength was 16.99%. In comparison, the average value of the 28 d strength was only 10.41%, indicating that the addition of PPF made a relatively significant contribution to the early strength of recycled brick aggregate concrete, mainly because of the large amount of PPF, small spacing, and fineness, which could significantly inhibit the early non-oriented plastic cracking of concrete, improving its early compressive strength.

### 3.2. Compressive Strength of Axial

**Figure 5:**
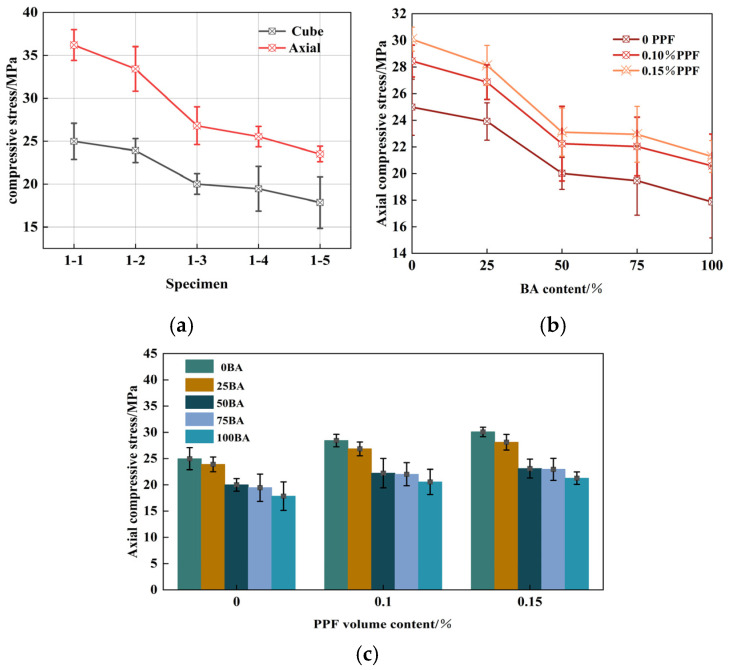
PPF recycled brick aggregate concrete axial compressive strength: (**a**) Comparison of cube and axial compressive stress (0 PPF); (**b**) Effects of BA content (0, 0.1%, 0.15% PPF); (**c**) Effects of PPF volume content.

Figure 5 shows the 28 d axial compressive strength of PPRAC. The variation trend of the axial compressive strength was the same as that of the cube compressive strength. The strength decreased rapidly when the BA content was 25–50%. When the replacement rate of recycled aggregate was 25%, 50%, 75%, and 100%, the 28 d axial compressive strength gradually decreased by 4.28%, 12.11%, 7.42%, and 8.21%. When 0.1% PPF was added, the increase was 14.01%, 12.35%, 11.12%, 13.20% and 15.20%, respectively. Compared with 0.1% PPF, the axial compressive strength of the 28-day cylinder with 0.15% PPF increased only by 5.78%, 4.72%, 3.92%, 4.18%, 3.45%. Due to the “wall effect” of the aggregate, excess fibers were aligned on and around the surface of the coarse aggregate, resulting in defects that weakened the reinforcement of the fibers.

The improvement effect of PPF on the recycled brick aggregate concrete axial compressive strength was better than that of the cube compressive strength. This can be explained by the fact that the two ends of the axial compressive strength specimen were less constrained by the loading equipment when the axial compressive strength specimen failed, and PPF could play the role of a crack bridge more effectively, thus improving the axial compressive strength.

### 3.3. Bending Strength

**Figure 6:**
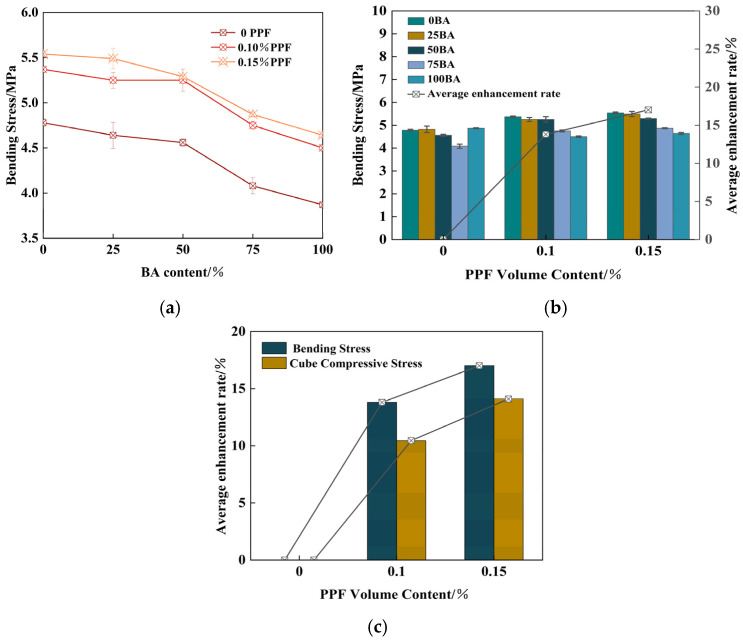
PPF recycled brick aggregate concrete bending strength: (**a**) Effects of BA content (0, 0.1%, 0.15% PPF); (**b**) Average enhancement rate with different PPF volume; (**c**) Average enhancement rate with cube compressive and bending stress.

Figure 6a shows that the flexural strength of recycled brick aggregate concrete decreased with an increase in the BA substitution rate. The 28 d bending strength of recycled brick aggregate concrete (0PPF) with 75% and 100% substitution rates decreased by 13.45% and 17.78%, respectively, compared with control group 1-1 (0BA 0PPF). The high RA substitution rate increased the number of new and old interfaces in the matrix, increasing the weak zone of cleavage. The fracture energy of the old interface was lower than that of the new interface, and the effect of load on the splitting tensile strength of the concrete with a high BA substitution rate was significant. The bending strength of recycled brick aggregate concrete with different BA substitution rates increased by 12.32%, 8.82%, 15.22%, 16.32% and 16.33%, respectively. This indicated that the energy consumption required for crack development under the bending action and the adverse effects of BA can be offset by the bridging action of PPF and the resistance of BA’s strength.

Figure 6b shows that the average bending strength of recycled brick aggregate concrete with PPF volume contents of 0.1% and 0.15% increased by 13.80% and 17.01%, respectively, compared with control group 1-1. Figure 6c shows the average increase rate of axial compressive strength and bending strength with the increase in PPF content. It can be observed that PPF contributed more to bending strength than compressive strength.

## 4. Mechanical Properties Index Calculation Method

We next investigated the relationship between the cube’s compressive strength and other mechanical properties, such as axial compressive strength, flexural strength, and elastic modulus. Where the elastic modulus was selected from the secant modulus between the longitudinal strain of 50 με and the strain corresponding to 40% of the peak stress on the stress–strain curve under uniaxial compression according to ASTM C469M [26], the mechanical indexes are shown in Table 8.

### 4.1. Axial Compressive Strength Calculation Formula

**Figure 7:**
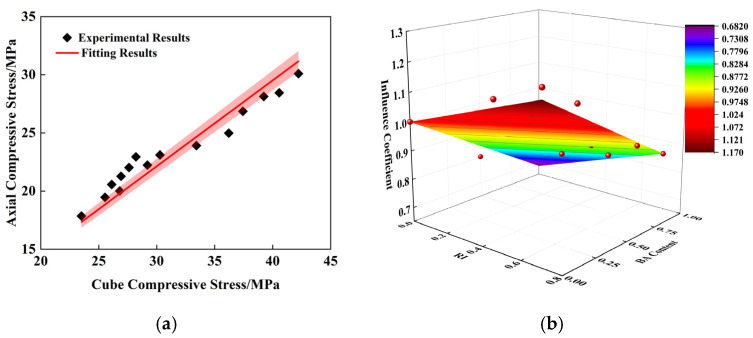
Fitting results of axial compressive strength: (**a**) Relation between axial compressive strength and cube compressive strength; (**b**) Influence coefficient ($\xi_{1}$).

The experimental data were processed by linear regression using Origin (2021) software. It can be seen from Figure 7a that the cube compressive strength ($f_{cu}$) and axial compressive strength ($f_{c}$) presented a strong linear relationship (R^2^ = 0.99); the regression result is shown in Formula (1). Considering the weakening effect of BA substitution rate on recycled concrete and the enhancement effect of PPF fiber content on it, the ratio of 1-1 between each group and the control group was defined as the influence coefficient ($\xi_{1}$).

Formula (2) was obtained by taking into account BA substitution rate and PPF reinforcement coefficient ($\mathrm{RI}=V_{\mathrm{PPF}}\times l_{\mathrm{PPF}}/d_{\mathrm{PPF}}$) and combining the calculation results of the influence coefficient (R^2^ = 0.920). The regression results are shown in Figure 7b. Finally, the conversion relationship between the cubic compressive strength and the axial compressive strength of PPF reinforced recycled brick aggregate concrete is shown in Formula (3).

(1) $$f_{c}=0.74f_{cu}$$

(2) $$\xi_{1}=1+0.240\mathrm{RI}-0.316\mathrm{BA}$$

(3) $$f_{c}=0.74f_{cu}\left(1+0.240\mathrm{RI}-0.316\mathrm{BA}\right)$$

### 4.2. Bending Strength Calculation Formula

**Figure 8:**
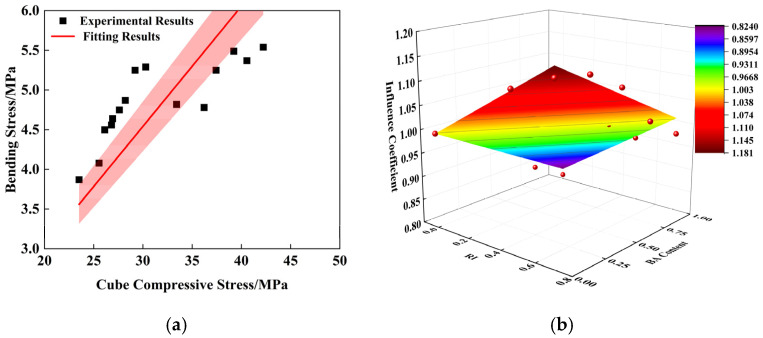
Fitting results of bending strength: (**a**) Relation between bending strength and cube compressive strength; (**b**) Influence coefficient ($\xi_{2}$).

Similarly, the linear regression method was used to fit the four-point bending test data; the results are shown in Figure 8a. The fitting formula is shown in Equation (4). The bending strength influence index ($\xi_{2}$) was obtained by fitting, considering the BA content and PPF enhancement coefficient.

The fitting results are shown in Figure 8b, where R^2^ = 0.917, indicating a good fit. The fitting formula is shown in Equation (5). The conversion Formula (6) between the bending strength ($f_{b}$) and the cubic compressive strength of PPRAC can be obtained by connecting the vertical (4) and Formula (5).

(4) $$f_{b}=0.15f_{cu}$$

(5) $$\xi_{2}=1+0.257\mathrm{RI}-0.176\mathrm{BA}$$

(6) $$f_{b}=0.15f_{cu}\left(1+0.257\mathrm{RI}-0.176\mathrm{BA}\right)$$

### 4.3. Elastic Modulus

PPRAC is a novel building material. Currently, no unified method exists to determine its elastic modulus (Ec) for reference. This study selected several ordinary concrete elastic modulus calculation formulas (Table 9) to compare and analyze the experimental data.

**Figure 9:**
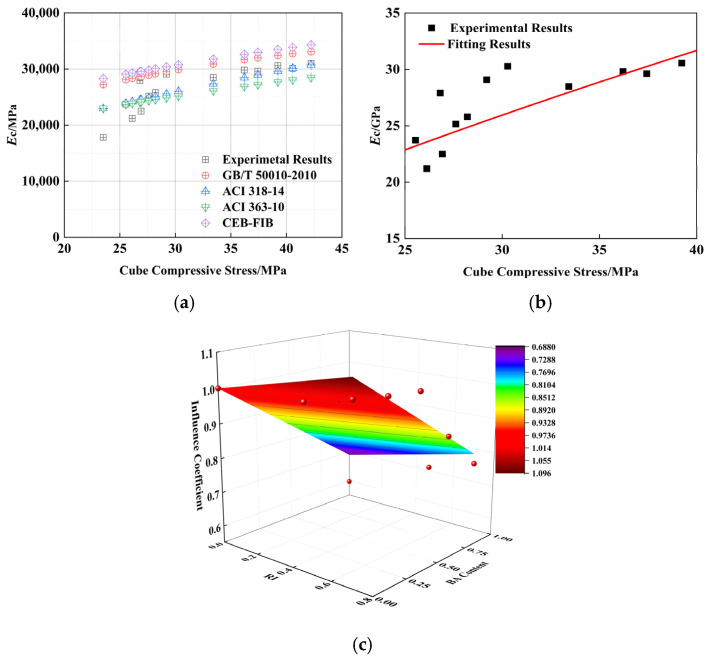
Fitting results of elastic modulus: (**a**) Comparison of different calculation methods [27,28,29,30]; (**b**) Relation between bending strength and cube compressive strength; (**c**) Influence coefficient ($\xi_{3}$).

A comparison of the calculation results is shown in Figure 9a. The calculation method in the form of a power function adopted from the literature [27,28,29,30] agreed with the experimental data in this study. Therefore, a regression analysis of the experimental data was performed using the power function; the results are shown in Figure 9b, R^2^ = 0.89. Consistent with the process in the paper, prediction Equation (8) was established considering the BA content and fiber reinforcement index RI. The fitting results are shown in Figure 9c, and the fitting coefficient R^2^ = 0.85. The conversion Formula (9) between the elastic modulus ($f_{e}$) and the cubic compressive strength of PPF reinforced recycled brick aggregate concrete could be obtained by connecting the vertical (7) and Formula (8).

(7) $$E_{c}=2450f_{cu}^{0.69}$$

(8) $$\xi_{3}=1+0.135\mathrm{RI}-0.310\mathrm{BA}$$

(9) $$E_{c}=2450f_{cu}^{0.69}\left(1+0.135\mathrm{RI}-0.310\mathrm{BA}\right)$$

## 5. Carbon Emission Analysis of PPF Reinforced Recycled Brick Aggregate Concrete

### 5.1. Carbon Emission Analysis

Life cycle assessment (LCA), as a whole life cycle assessment method, has been applied in various industries. LCA compiles and evaluates a product system’s input, output, and potential environmental impact during its life cycle. Its primary purpose is to reduce energy consumption and environmental emissions in the system’s production process and improve system activities’ socio-economic and environmental performance throughout the life cycle. It has become an essential tool for resource management and sustainable development assessments. It has been widely used in various fields [31,32,33]. The life cycle method is a “bottom-up” accounting method which is widely used in the carbon emission accounting of products or active processes.

Relevant studies have proved [34] that the CO_2_ resulting from producing concrete raw materials comprises a significant proportion of the total for concrete manufacturing, i.e., as high as 84~93%. In this study, the LCA method was used to calculate the carbon emissions in the production process of each material of PPF-reinforced recycled brick aggregate concrete. Reference [35] was combined with Equation (10) to calculate the carbon emissions of each category in the study. In formula (10), C_sc_ is the carbon emission in the production stage of raw materials, *M_i_* is the consumption of the i type of raw materials, and *F_i_* is the carbon emission factor of the i raw material. The calculation results are listed in Table 10.

(10) $$C_{sc}=\sum_{i=1}^{n}M_{i}F_{i}$$

**Figure 10:**
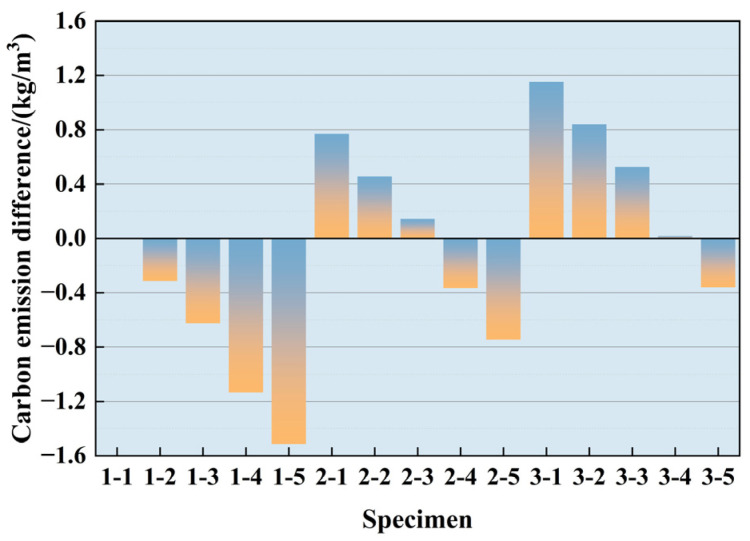
Carbon emission difference of each experimental group.

Table 10 shows that the CO_2_ emission of control group 1-1 was 362.0201 kg, of which the CO_2_ produced by cement accounted for 98.88% of the total. Figure 10 shows that with the incorporation of BA, a specific reduction in carbon emissions was not apparent, and the effect of energy saving and emission reduction solely relying on recycled aggregate is limited. In addition, although incorporating PPF can enhance the strength of concrete, the carbon emissions will also increase. Therefore, it is necessary to consider the strengths and environmental benefits comprehensively when designing a mix ratio.

### 5.2. Environmental Benefit Analysis

**Figure 11:**
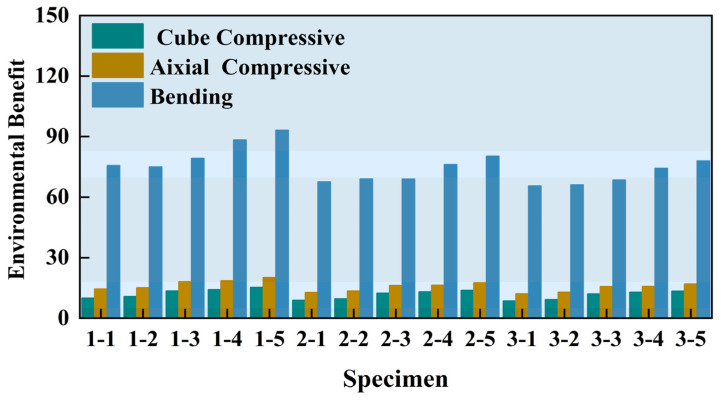
Results of environmental benefits of ppf reinforced recycled brick aggregate concrete.

According to the carbon emission analysis in the above section, although adding PPF can effectively enhance the strength of concrete, the environmental benefits are also reduced. At present, although the recycling of agricultural waste and food waste made of fiber has better environmental benefits, such fibers often need to be processed by special means, which seriously restricts their application [35]. Therefore, the use of polypropylene fiber is inevitable. To comprehensively consider the strength and environmental benefits, the environmental benefit coefficient was calculated by Equation (11), where *F_i_* represents cube compressive strength, axial compressive strength, and bending strength respectively. The calculation results are shown in Table 11, and Figure 11 shows the trend of environmental benefits for each group.

(11) $$F_{i}I_{co_{2}}=\frac{C_{SC}}{f_{i}}$$

It was found that the environmental benefits index of groups 2-1, 2-2, 3-1, and 3-2 was low; considering the mechanical properties of concrete and carbon emissions, the BA substitution rate should not exceed 50%. With an increase in PPF content, the environmental benefits of PPF-reinforced brick aggregate recycled concrete also increased. However, its impact on the environmental benefits was significantly less than the strength benefits it provided. Therefore, considering the joint impact of the replacement rate of recycled brick solid waste and the enhancement of PPF, the strength and environmental benefits of the 2–3 groups of specimens were the best.

## 6. Conclusions

This study investigated the macroscopic mechanical properties of PPRAC, explored the effects of the replacement rate of recycled aggregate and the volume content of PPF fiber on it, built a mechanical parameter calculation model based on the above, and finally combined the life cycle evaluation method to reach the following conclusions:

(1) With an increase in the substitution rate of BA, the strength of PPRAC initially displayed a trend of slow decline, followed by a rapid decline with 50% content serving as the critical point. This trend was manifested in the cube compressive strength, axial compressive strength, and bending strength. It is preliminarily concluded that the replacement rate of BA should not exceed 50%. The PPF fiber effectively mitigated the weakening of the macroscopic mechanical properties of concrete caused by the initial defects of BA through the bridging effect, which was more pronounced in the bending strength.
(2) PPRAC is a new type of multiphase composite material, and its strength calculation relationship is still unknown. Based on the experimental data in this paper and by referring to the research results of others, the transformation formula between the axial compressive strength, bending strength, elastic modulus, and cubic compressive strength considering the reduction of BA content and the enhancement of PPF were constructed. The R^2^ of the conversion formulas were all around 0.9, the regression effect was good, and the equation had strong applicability.
(3) As the substitution rate of BA increased, the carbon emissions of PPRAC declined. Nevertheless, the reduction effect was not significant, and the effect of reducing carbon emissions by replacing coarse aggregate was limited. With the addition of PPF, the carbon emissions of recycled brick aggregate concrete rose, but the environmental benefit was still considerable, compared with the strength benefit. Considering the recovery rate of recycled aggregate and the environmental benefit of the material, the PPF fiber-reinforced recycled brick aggregate concrete with 0.1% PPF fiber volume content and 50% BA substitution rate possesses good mechanical properties and should be further promoted and utilized.

## Figures and Tables

**Table 1:** Basic physical properties of coarse aggregate.

Aggregate Class	Particle Size Range /mm	Mud Content /%	Water Absorption/%	Bulk Density/(kg/m^3^)	Apparent Density/(kg/m^3^)
BA	5~20	1.3	15.5	1015	2160
NA	5~20	1.1	1.1	1450	2690

**Table 2:** PPF performance index.

Fiber Class	Length /mm	Diameter /µm	Volume Density/(g/cm^3^)	Tensile Stress /MPa	Elongation /%
PPF	15	32	0.89	415	15

**Table 3:** Main chemical components of Portland cement type P·O 42.5.

C_3_S	C_2_S	C_4_AF	C_3_A	f-MgO	f-CaO	Else
60.2	19.4	8.5	7.1	2.0	0.7	2.1

**Table 4:** Physical properties of P·O 42.5 cement.

Standard Consistency Water Consumption/%	Stability	Heat Loss /%	Density /(kg/m^3^)	Solidification Time/min		Flexural Strength/MPa	
				Initial	Final	3 d	28 d
28.2	eligible	3.75	3110	165	305	5.4	8.8

**Table 5:** Fine aggregate properties.

Capacity/(kg/m^3^)	Water Absorption/%	Apparent Density/(kg/m^3^)
1655	0.51	1689

**Table 6:** C30 concrete mix ratio-1(%).

Number	PPF	NA	BA
1-1	0	100	0
1-2	0	75	25
1-3	0	50	50
1-4	0	25	75
1-5	0	0	100
2-1	0.10	100	0
2-2	0.10	75	25
2-3	0.10	50	50
2-4	0.10	25	75
2-5	0.10	0	100
3-1	0.15	100	0
3-2	0.15	75	25
3-3	0.15	50	50
3-4	0.15	25	75
3-5	0.15	0	100

**Table 7:** C30 concrete mix ratio-2.

Number	Water/(kg/m^3^)	Cement/(kg/m^3^)	NA (5–20mm)/(kg/m^3^)	BA (5–20 mm)/(kg/m^3^)	Natural Medium Sand/(kg/m^3^)	PPF/%
1-1	195.0	487.0	1138	0	613.0	0
1-2	195.0	487.0	853.5	284.5	613.0	0
1-3	195.0	487.0	569	569	613.0	0
1-4	195.0	487.0	284.5	675	613.0	0
1-5	195.0	487.0	0	900	613.0	0
2-1	195.0	487.0	1138	0	613.0	0.10
2-2	195.0	487.0	853.5	284.5	613.0	0.10
2-3	195.0	487.0	569	569	613.0	0.10
2-4	195.0	487.0	284.5	675	613.0	0.10
2-5	195.0	487.0	0	900	613.0	0.10
3-1	195.0	487.0	1138	0	613.0	0.15
3-2	195.0	487.0	853.5	284.5	613.0	0.15
3-3	195.0	487.0	569	569	613.0	0.15
3-4	195.0	487.0	284.5	675	613.0	0.15
3-5	195.0	487.0	0	900	613.0	0.15

**Table 8:** Experimental results.

Number	Cube Compressive Stress/MPa	Axial Compressive Stress/MPa	Bending Stress/MPa	Elastic Modulus/GPa
1-1	36.21	24.98	4.78	29.82
1-2	33.42	23.91	4.82	28.49
1-3	26.81	20.01	4.56	27.91
1-4	25.54	19.46	4.08	23.73
1-5	23.51	17.86	3.87	17.80
2-1	40.56	28.45	5.37	30.12
2-2	37.43	26.86	5.25	29.63
2-3	29.20	22.24	5.22	29.09
2-4	27.61	22.03	4.75	25.16
2-5	26.12	20.57	4.5	21.20
3-1	42.22	30.09	5.54	31.02
3-2	39.23	28.13	5.49	30.57
3-3	30.28	23.11	5.29	30.29
3-4	28.21	22.95	4.87	25.80
3-5	26.92	21.28	4.64	22.50

**Table 9:** Expressions between compressive strength and elastic modulus of concrete.

Reference	Equation	Scope of Application
GB/T 50010-2010 [27]	$E_{c}=\frac{10^{5}}{2.2+34.7/f_{c}}$	$15\leq f_{c}\leq 80\mathrm{MPa}$
ACI 318-14 [28]	$E_{c}=4730\sqrt{f_{c}}$	$f_{c}\leq 40\mathrm{MPa}$
ACI 363-10 [29]	$E_{c}=3320\sqrt{f_{c}}+6900$	$21\leq f_{c}\leq 83\mathrm{MPa}$
CEB-FIB [30]	$E_{c}=9979.4f_{c}^{0.33}$	$f_{c}\leq 80\mathrm{MPa}$

**Table 10:** The CO_2_ emission factors of different materials (kg/m^3^).

Material	NA	BA	Sand	PPF	Water	PC	Total
CO_2_ emission factors	0.0022	0.0011	0.0025	0.8540	0.0390	0.7350	-
1-1	2.5036	0	1.5325	0	0.0390	357.9450	362.0201
1-2	1.8777	0.3130	1.5325	0	0.0390	357.9450	361.7072
1-3	1.2518	0.6259	1.5325	0	0.0390	357.9450	361.3942
1-4	0.6259	0.7425	1.5325	0	0.0390	357.9450	360.8849
1-5	0	0.9900	1.5325	0	0.0390	357.9450	360.5065
2-1	2.5036	0	1.5325	0.7686	0.0390	357.9450	362.7887
2-2	1.8777	0.3130	1.5325	0.7686	0.0390	357.9450	362.4758
2-3	1.2518	0.6259	1.5325	0.7686	0.0390	357.9450	362.1628
2-4	0.6259	0.7425	1.5325	0.7686	0.0390	357.9450	361.6535
2-5	0	0.9900	1.5325	0.7686	0.0390	357.9450	361.2751
3-1	2.5036	0	1.5325	1.1529	0.0390	357.9450	363.1730
3-2	1.8777	0.3130	1.5325	1.1529	0.0390	357.9450	362.8601
3-3	1.2518	0.6259	1.5325	1.1529	0.0390	357.9450	362.5471
3-4	0.6259	0.7425	1.5325	1.1529	0.0390	357.9450	362.0378
3-5	0	0.9900	1.5325	1.1529	0.0390	357.9450	361.6594

**Table 11:** Results of environmental efficiency.

Specimen	$F_{\mathrm{cu}}I_{{\mathrm{co}}_{2}}$	$F_{c}I_{{\mathrm{co}}_{2}}$	$F_{b}I_{{\mathrm{co}}_{2}}$
1-1	9.9978	14.4924	75.7364
1-2	10.8231	15.1279	75.0430
1-3	13.4798	18.0607	79.2531
1-4	14.1302	18.5450	88.4522
1-5	15.3342	20.1851	93.1541
2-1	8.9445	12.7518	67.5584
2-2	9.6841	13.4950	69.0430
2-3	12.4028	16.2843	68.9834
2-4	13.0986	16.4164	76.1376
2-5	13.8314	17.5632	80.2834
3-1	8.6019	12.0696	65.5547
3-2	9.2496	12.8994	66.0947
3-3	11.9732	15.6879	68.5344
3-4	12.8337	15.7751	74.3404
3-5	13.4346	16.9953	77.9438

## Data Availability

The datasets used and/or analyzed during the current study are available from the corresponding author on reasonable request.
